# 1.5T MR-Guided Daily-Adaptive SBRT for Prostate Cancer: Preliminary Report of Toxicity and Quality of Life of the First 100 Patients

**DOI:** 10.3390/jpm12121982

**Published:** 2022-11-30

**Authors:** Filippo Alongi, Michele Rigo, Vanessa Figlia, Luca Nicosia, Rosario Mazzola, Niccolò Giaj Levra, Francesco Ricchetti, Giovanna Trapani, Giorgio Attinà, Claudio Vitale, Edoardo Pastorello, Antonio De Simone, Davide Gurrera, Stefania Naccarato, Gianluisa Sicignano, Ruggero Ruggieri, Francesco Cuccia

**Affiliations:** 1 Advanced Radiation Oncology Department—IRCCS Sacro Cuore Don Calabria Hospital, 37024 Negrar di Valpolicella, Italy; 2 University of Brescia, 25121 Brescia, Italy

**Keywords:** MR-linac, stereotactic body radiotherapy, prostate cancer, daily-adaptive RT

## Abstract

**Purpose:** The present study reports the preliminary outcomes in terms of adverse events and quality of life in the first 100 patients treated with 1.5T MR-guided daily-adaptive stereotactic body radiotherapy for prostate cancer. **Methods:** From October 2019 to December 2020, 100 patients, enrolled in a prospective study, received MR-guided SBRT for prostate cancer. Rectal spacer insertion was optional and administered in 37 patients. In total, 32 patients received androgen deprivation therapy in accordance with international guidelines. A prospective collection of data regarding toxicity and quality of life was performed. **Results:** The median age was 71 years (range, 52–84). The median total dose delivered was 35 Gy (35–36.25 Gy) in five sessions, either on alternate days (n = 25) or consecutive days (n = 75). For acute toxicity, we recorded: seven cases of acute G2 urinary pain and four cases of G2 gastrointestinal events. The median follow-up was 12 months (3–20), recording three late G2 urinary events and one G3 case, consisting of a patient who required a TURP 8 months after the treatment. For gastrointestinal toxicity, we observed 3 G ≥ 2 GI events, including one patient who received argon laser therapy for radiation-induced proctitis. Up to the last follow-up, all patients are alive and with no evidence of biochemical relapse, except for an M1 low-volume patient in distant progression two months after radiotherapy. QoL evaluation reported a substantial resolution of any discomfort within the second follow-up after radiotherapy, with the only exception being sexual items. Notably, after one year, global health items were improved compared to the baseline assessment. **Conclusions:** This study reports very promising outcomes in terms of adverse events and QoL, supporting the role of 1.5T MR-guided SBRT for prostate cancer. To date, this series is one of the first and largest available in the literature. Long-term results are warranted.

## 1. Introduction

Prostate cancer is one of the most frequently diagnosed tumors in the male population, accounting for about 23% of cases in Europe in the last year [1].

For localized disease, external beam radiotherapy (EBRT) is considered the preferred non-invasive curative alternative to surgery [2].

Due to the very low alpha beta ratio of prostate cancer, hence with a higher sensitivity to higher doses per fraction, large amounts of literature data of hypofractionated schedules supported the recommendation of >2 Gy/fraction regimens being added into clinical guidelines both in Europe and US [3,4,5,6].

In the last years, a growing number of studies in the literature have explored the possibilities of extreme hypofractionated schedules with stereotactic body radiotherapy (SBRT), reported as a therapeutic strategy able to provide promising results, both in terms of tolerance and clinical outcomes [7,8].

Specifically in this setting, the need to improve dose delivery accuracy becomes more relevant due to the inter- and intra-fractional variability of both the target and nearby healthy structures. In this setting, the availability of hybrid magnetic resonance (MR)-linacs may represent a potential game changer for the treatment of prostate cancer [9,10]

The on-board MR imaging allows clinicians to increase the quality of image guidance (IG) compared to conventional CT-based IGRT, by improving the soft tissue contrast visualization of the pelvic anatomy. Consequently, prostate volumes are supposed to be identified in smaller volumes with a lower exposure of the healthy structures [11].

Furthermore, these hybrid machines allow the daily adaptation of the plan based on real-time anatomy, taking into account daily variability in the volume, shape and size of both target volumes and nearby healthy structures. Interestingly, the refined accuracy in volume identification and with the increased confidence in the IGRT phase results for the delivery phase, the patient can be spared the need for fiducials and invasive procedures [12].

All these advantages are supposed to lead to a favorable impact in terms of toxicity incidence, as hypothesized by the currently ongoing MIRAGE trial, aiming to compare the rates of acute G ≥ 2 genitourinary (GU) toxicity between MR-guided SBRT and conventional CT-based SBRT [13].

As a drawback, MR-linacs may be burdened with potentially longer treatment session times, which may affect intra-fraction organ motion. Furthermore, this theoretically requires a higher level of compliance from the patient, highlighting the need for a more accurate patient selection process in this specific setting [14].

To date, two main systems are commercially available, 1.5T MR-linac Elekta Unity (Elekta, Stockholm, Sweden) and 0.35 T MR-linac Viewray MRIdian (Viewray, Mountain View, CA, USA). Due to the recent age of this technology, there are few experiences reporting the preliminary outcomes of MR-guided SBRT for prostate cancer, with encouraging results in terms of safety and tolerance [15,16,17].

The ongoing prospective PRISM and MOMENTUM studies will provide further evidence in support of this technology for prostate SBRT [18,19].

In October 2019, in our Department, we started our clinical activity with the 1.5T MR-linac Unity Elekta (Stockholm, Sweden). This machine couples a 7 MV flattening filter free linear accelerator with an on-board 1.5T MR imager for IGRT. Since the beginning of our activity, all patients treated with this device have been enrolled in a prospective observational study approved by the local Ethical Committee (MR-Linac n° 23.748), having as a primary endpoint the evaluation of the feasibility and safety of the 1.5T MR-guided daily-adaptive approach. In this series, we report the preliminary results concerning the early toxicity and patient-reported outcomes (PROMs) of the first 100 patients who have been treated with MR-guided SBRT for prostate cancer. 

## 2. Methods

The present study was approved by the internal Ethical Committee on April 2019 (MRI/LINAC n°23.748). We report the initial results of the first 100 patients treated with MR-guided SBRT for prostate cancer. 

Inclusion criteria for the study were as follows: age > 18 years, ECOG PS ≤ 2 or Karnofsky index > 70%, histological diagnosis of prostate adenocarcinoma, including both localized cT1-cT3b disease and low-volume M1 disease according to the CHARTEED definition (delivering SBRT only to the prostate); no prior prostate surgery except for transurethral resection (TURP) with a minimum interval of 6 months from the start of SBRT), no other oncological diagnoses within the last 5 years, International Prostate Symptom Score (IPSS) 0–15. Concurrent androgen deprivation therapy (ADT) was prescribed depending on risk group. 

The exclusion criteria were: a prostate volume ≥ 80 cc, previous pelvic radiotherapy, general MRI contraindications (pacemakers or implanted cardioverter-defibrillators, brain stimulators, cochlear prosthetics or other metal devices or claustrophobia), inability to obtain written informed consent. 

The spacer hydrogel insertion was proposed as an optional tool to patients with localized prostate cancer and performed in 37 patients. The procedure was performed by the urologist. In these patients, SBRT start was scheduled to take place within 3 weeks after the insertion procedure.

### 2.1. Radiotherapy Procedures

Patient positioning and preparation required a comfortably full bladder (i.e., 500 cc of water 15–20 min earlier) and empty rectum (using a fleet enema 2 h before the procedure). A 3 mm slice thickness pelvis CT scan was performed for all patients for dose calculation. By means of KneeSTEP and FeetSTEP devices (Elekta, Stockholm, Sweden), the same positioning was used for a T2-weighted gradient echo pelvis MRI, including a coil anteriorly positioned for imaging acquisition. 

For low-risk disease, the target volume delineation was as follows: the clinical target volume (CTV) equal to the prostate gland; for intermediate-, high-risk and M1 low-volume patients the CTV also included the whole seminal vesicles (SV). 

The planning target volume (PTV) consisted of the CTV plus a 5 mm margin in all directions, except for the posterior direction where a 3 mm margin was applied, according to the literature data. As organs at risk (OARs), we contoured rectum, bladder and prostatic urethra, penile bulb and femurs. 

Regarding radiotherapy treatment, our 5 fractions SBRT schedule was set to deliver a total dose of 35 Gy to all the patients with low-risk disease, and a total dose of 36.25 Gy to all patients with intermediate-risk disease. The latter schedule was also applied for patients with a high risk of disease who refused moderate hypofractionated regimens due to logistical reasons. For the same reason, for low-volume M1 patients, the SBRT schedule consisted of 35 Gy in 5 consecutive fractions. When equivalent doses in 2 Gy per fraction (EQD2) were reported, total delivered doses ranged between 70–90.6 Gy assuming an α/β ratio between 3 and 1.5 Gy for prostate cancer [20].

Treatment planning was performed in order to guarantee a minimum 95% of the PTV to be covered by at least 95% of the prescribed dose, with ≤2% of the PTV covered by 107% of the prescribed dose.

Baseline plan optimization was performed with intensity modulated radiotherapy (IMRT) consisting of 16 static fields in step-and-shoot approach. The same strategy was applied daily for online ATS sessions. For both offline planning and daily sessions, we used these peer-reviewed literature constraints for the OARs: D1cc ≤ 35Gy, V32Gy ≤ 5%, V28Gy ≤ 10%, V18Gy ≤ 35%, for rectum; D1cc ≤ 35Gy for urethra planning organ at risk volume (PRV) and entire bladder [21].

Two alternative options are available to perform daily-adaptive radiotherapy with Elekta Unity: the ‘adapt-to-position’ (ATP) and ‘adapt-to-shape’ (ATS) methods. The first consists of isocenter position update prior to every session and it does not include the daily re-delineation. In the ATS workflow, the physician performs a complete delineation of all the structures (OARs and target volumes) on daily MRI; based on these new contours, a new re-planning is calculated based on the real-time anatomy.

In more detail, as described in a previous study [22], a T2-weighted MRI (pre-MRI) is performed and fused with baseline imaging. The physician edits the original contours on the pre-MRI, as necessary. A further MRI is performed to verify any change in volume or shape of bladder and rectum. If the deformations are not negligible, the procedure starts again from the beginning, otherwise the delivery proceeds after the acquisition of a coronal and sagittal cine MRI to check organ motion. 

### 2.2. Toxicity and Quality of Life Assessment

A prospective collection of acute and late adverse events was performed based on the Common Terminology Criteria for Adverse Events (CTCAE v5.0). The follow-up program started with the first follow-up scheduled after 2 months from the end of treatment, and subsequently every 90 days for the first year. 

With the same periodicity, quality of life (QoL) assessment was performed at baseline, at the end of SBRT and then at every follow-up visit with the following questionnaires:

– International Prostatic Symptoms Score (IPSS)
– EORTC Quality of Life Questionnaire-Core 30 (EORTC QLQ-C30) and EORTC QLQ-PR25
– Expanded Prostate Cancer Index Composite-26 (EPIC-26)
– International Consultation on Incontinence Questionnaire—Short Form (ICIQ-SF)
– International Index of Erectile Function—5 (IIEF-5)

### 2.3. Statistical Analysis

Descriptive data were collected for baseline patients’ characteristics. Survival estimates were performed using Kaplan–Meier method, assuming as statistically significant a *p*-value ≤ 0.05. PROMS assessment was calculated using the Wilcoxon signed rank test. All statistical analyses were carried out using SPSS (version 20.0; IBM, Armonk, NY, USA).

## 3. Results

From October 2019 to December 2020, 100 patients with a median age of 71 years (range, 52–84) were treated with 1.5T MR-guided daily-adaptive radiotherapy. The median IPSS was 3 (0–15). Risk groups were as follows: 34 low-risk, 29 favorable intermediate-risk, 31 unfavorable intermediate-risk, 2 high-risk and 4 low-volume M1 patients. Androgen deprivation therapy was administered in 32 patients (5 unfavorable intermediate-risk patients refused hormone therapy). SBRT was delivered over five sessions for a median total dose of 35 Gy (35–36.25 Gy) either on consecutive (n = 75) or alternate days (n = 25). The mainly adopted workflow was ATS in 480 out of 500 sessions as we considered this workflow more accurate for prostate SBRT delivery), for a median treatment length of 40 min (33–83 min). The median PTV volume was 105.8 cc (13.98–196.4 cc).

A brief summary of patients’ characteristics is reported in Table 1.

### 3.1. Acute Toxicity

A total of 7% of G2 events of acute genitourinary events occurred, consisting of five cases of pain and two patients who required a short-term catheterization due to urethral stenosis. For gastrointestinal adverse events, we recorded a 4% (n = 4) incidence of G2 events consisting of rectal tenesmus or proctitis. All these events were registered after an average time of 30 days from radiotherapy. Specifically for the two cases of urethral stenosis, no significant baseline conditions or dosimetric parameters were found to be potentially predictive of toxicity. In one patient, the catheterization was positioned after the first session treatment and immediately removed the day after with no significant consequences during the treatment and the follow-up. The other patient required 3 days of catheterization after the end of the treatment with a subsequent complete resolution of the symptoms.

We also performed a subgroup analysis comparing the outcomes of patients who received the rectal hydrogel spacer with patients who did not. Specifically for the spacer cohort, we detected three cases of acute G2 GI toxicity and three cases of acute G2 GU toxicity. Otherwise, in the no-spacer sample, we observed four cases of G ≥ 2 GU events and one G2 GI event. A detailed list of acute and late toxicity is reported in Table 2. 

### 3.2. Late Toxicity

After a median follow-up of 12 months (3–20 months), three late G2 genitourinary events occurred, consisting of urinary pain; a single G3 GU toxicity was recorded in a patient who developed a urethral stenosis 8 months after SBRT, requiring a TURP. Regarding gastrointestinal toxicity, three patients developed G ≥ 2 GI proctitis, with one G3 case requiring an argon laser treatment. Up to the last follow-up, all patients are under biochemical control except for one M1 low-volume patient in distant progression from two months after the end of radiotherapy.

### 3.3. PROMs Assessment

A preliminary QoL assessment revealed a general trend toward a complete return to baseline values by the second follow-up, with the exception of sexual function domains that remained impaired after SBRT treatment. Interestingly, global health domains of the QLQ-C30 questionnaire reported a statistically significant improvement during the follow-up assessments (Supplementary Materials)

– IPSS: Median IPSS scores were 4.7 at baseline, 9.7 at the end of MR-guided adaptive SBRT and 6.3 at the first follow-up. We reported a significant increase in the IPSS score at the end of radiation treatment (*p*: 0.001), with a return to similar baseline values by the second follow-up (*p*: 0.81).
– EORTC-QLQ-PR25: Only the urinary symptoms, incontinence aid and bowel symptoms were affected to a statistically significant magnitude after treatment and at the first follow-up, with a return to baseline values at the second follow-up assessment.
– ICIQ-SF: a significant variation was only recorded at the end of treatment time, with a return to baseline values at the first follow-up
– IIEF-5: A statistically significant score reduction was registered at the post-RT time and at the first follow-up, with a return to baseline conditions at the second follow-up time.
– EPIC-26: Urinary symptom items were significantly affected after the end of treatment and at the first follow-up evaluation. The sexual functioning domain was significantly affected at each assessment time, up to the 12 months evaluation.
– EORTC-QLQ-C30: Financial difficulties, pain, insomnia and physical and role functioning were affected at the initial post-SBRT assessments with a return to baseline values by the second follow-up; interestingly, global health status, and emotional and social functioning domains reported improved scores of a statistically significant magnitude compared to baseline values at the third follow-up assessment.

## 4. Discussion

To the best of our knowledge, the present study reports the first and largest experience regarding the preliminary outcomes of patients who received 1.5T MR-guided SBRT for prostate cancer. To date, a similar experience was first reported by Bruynzeel et al. [15] concerning a cohort of 101 patients who were treated with 0.35T MR-guided SBRT collecting encouraging preliminary results in terms of safety and tolerability. More specifically, they reported acute GI G ≥ 2 events in 3% of cases, and acute GU G ≥ 2 effects in 19.8%, including a 5.9% incidence of G3 toxicity according to the RTOG criteria. 

More recently, the same authors updated their results with a longer follow-up and a more detailed QoL analysis, reporting a substantial recovery of all the domains after one year [16].

A further experience of 0.35T MR-guided SBRT has been reported by Uguerler et al. in a series of 50 patients, collecting acute G2 GU toxicity in 36% of cases, with no acute G ≥ 2 GI events [23].

In our series, we reported excellent results in terms of acute and late toxicity incidence, with acute G ≥ 2 GU and GI events respectively in 7% and 4% of cases. The smaller incidence of acute G ≥ 2 GU events in our series may be related to the relatively lower prescription dose of 35 Gy/5 fractions in a proportion of patients, compared to the abovementioned studies in which only patients who received 36.25 Gy in five fractions were evaluated. Notably, compared to these 0.35T experiences, we applied a wider margin of 5 mm in all directions except for 3 mm posteriorly, and we stated as an exclusion criterion a prostate volume larger than 80 cc vs. 90 cc. of the 0.35T experiences. 

Unlike the abovementioned studies, in our series, we also included patients who received the hydrogel rectal spacer insertion prior to the SBRT treatment. In a subgroup analysis, we reported no significant differences with patients who did not receive the spacer, and, interestingly, the procedure did not affect QoL scores. This is in agreement with a previous study of our department, in which the administration of the rectal spacer gel did not result in a detrimental impact on PROMs scores. Concerning the role of the hydrogel on late toxicity, it is probable that longer follow-up data will provide more information on rectal toxicity patterns, given the greater propensity of GI events to occur later [24,25]

In a further experience recently published by Magli et al. about the toxicity profile of a three-fractions schedule delivered with conventional linac, in which all patients received both the hydrogel spacer and 3–4 fiducial markers positioning, the authors detected a significative worsening of the IPSS and ICIQ-SF scores after 12 months [26].

Concerning the isolated cases of two patients requiring catheterization, we did not find any particular baseline condition potentially predictive for an increased risk of toxicity. Furthermore, one of the two patients fully recovered from the catheterization after the first fraction and then completed the treatment with no interruptions. Moreover, the subsequent follow-up visits were negative for any other adverse event. 

We recorded only one case of late G3 GU toxicity with a patient requiring a TURP after SBRT due to the evidence of severe urethral stenosis. Additionally in this case, we failed to identify any potential risk factor for an increased incidence of adverse events. 

This is also confirmed by our QoL analysis, in which no significant impact was detected in the follow-up questionnaires when compared to baseline scores. 

Only the sexual functioning domain maintained a statistically significant decrease on the EPIC-26 questionnaire, although this not confirmed by the IIEF-5. The discrepancy between these two different QoL tests suggests the need for a longer follow-up to further evaluate the impact of the treatment on sexual activity. 

A transient decline in some items concerning urinary symptoms was observed after the end of treatment, with a full recovery by the first or second follow-up. 

These data have a double favorable impact: first of all, they reflect the promising toxicity pattern of our experience, with minimal G ≥ 2 events incidence. Secondly, despite the longer treatment session length theoretically affecting the patient’s compliance and consequently having a detrimental impact on QoL, our PROMs assessment reported no statistically significant effect on living condition. Further confirmation of this favorable impact is shown by the findings regarding the EORTC-QLQ-C30 in which a statistically significant improvement in the scores of the global health, social and role functioning domains was registered, highlighting the encouraging perception of the patient towards the treatment both in terms of initial outcomes and toxicity patterns. 

These findings were also anticipated in a previous report of our department regarding the first 25 patients enrolled in the study, in which no significant variations in terms of PROMs scores were detected [17].

The confirmation of the safety and excellent tolerability of this technology reinforce the huge potential of MR-linacs for prostate SBRT, as it may be the optimal choice for hypothesizing further steps forward, for example, in terms of single fraction treatments or boosting the dominant intraprostatic lesion or sexual structures preservation [14].

This study has some limitations: first of all, the relatively short follow-up may affect late toxicity evaluation, although previous experiences of prostate SBRT report a substantial tendency of late toxicity to remain stable after one year of follow-up [8,27].

Moreover, this series of 100 consecutive patients collects a quite inhomogeneous sample, also including patients who received 1.5T MR-guided SBRT with a hydrogel rectal spacer. Nonetheless, in a subgroup analysis, no relevant differences were observed when compared to patients who did not receive the gel prior to the SBRT treatment. Furthermore, in 32 cases, the longer length of androgen deprivation therapy may be a confounding factor for QoL assessment, although this preliminary PROMs analysis reports no significant differences compared to the majority of our study population. Lastly, a proportion of patients received concomitant ADT, which may have potentially biased the sexual function QoL assessment. 

Still, to the best of our knowledge, this is the first and largest experience that reports preliminary prospective data on toxicity and tolerability for 1.5T MR-guided SBRT for prostate cancer.

## 5. Conclusions

The present study supports the role of 1.5T MR-guided SBRT for the treatment of localized prostate cancer as a promising tool with excellent results in terms of acute toxicity and preliminary late toxicity. Notably, PROMS assessment revealed a negligible impact in terms of quality of life. More mature data will help clinicians to understand the real impact of this technique in terms of quality of life.

## Figures and Tables

**Table 1:** Patients’ characteristics.

Characteristic	Value
**Age**	71 years (range, 52–84)
**Risk Group**	
***Low Risk***	34
***Intermediate Risk (Favorable/Unfavorable)***	60 (29/31)
***High Risk***	2
***M1 low-volume***	4
**RT Schedule**	35 Gy/5 fx (n = 55); 36,25 Gy/5 fx (n = 45)
**Daily vs. Alternate Days**	75/25
**Hydrogel Spacer (y/n)**	37/63
**Androgen Deprivation Therapy (y/n)**	32/68
**ATP vs. ATS**	20/480
**Treatment Time**	40 min (33–83)

**Table 2:** Acute (up) and Late (down) toxicity patterns.

Genitourinary	G1		G2	
Urinary Tract Pain	15		5	
Urinary Urgency	11		-	
Urethral Stenosis	1		2	
**Gastrointestinal**	**G1**		**G2**	
Diarrhea	-		-	
Rectal Tenesmus	5		3	
Rectal Proctitis	4		1	
**Genitourinary**	**G1**	**G2**		**G3**
Urinary Tract Pain	10	2		-
Urinary Stenosis	3	1		1
Hematuria	-			-
**Gastrointestinal**	**G1**	**G2**		**G3**
Diarrhea	-	-		-
Rectal Tenesmus		2		-
Rectal Bleeding	-	-		1

## Data Availability

Not applicable.

## Supplementary Materials

The following supporting information can be downloaded at: https://www.mdpi.com/article/10.3390/jpm12121982/s1, Table S1—EORTC-QLQ-C30; Table S2—ICIQ-SF; Table S3—IIEF-5; Table S4—EORTC-QLQ-PR25; Table S5—EPIC-26; Table S6—IPSS.
