# Supplementary material for: 1.5T MR-Guided Daily-Adaptive SBRT for Prostate Cancer: Preliminary Report of Toxicity and Quality of Life of the First 100 Patients
Source: J Pers Med. 2022 Nov 30;12(12):1982. doi: 10.3390/jpm12121982 (PMC9785799; doi:10.3390/jpm12121982)
Supplement: Supplementary file 1 [file jpm-12-01982-s001.zip › jpm-2049558-supplementary.pdf]

Table S1 – EORTC-QLQ-C30

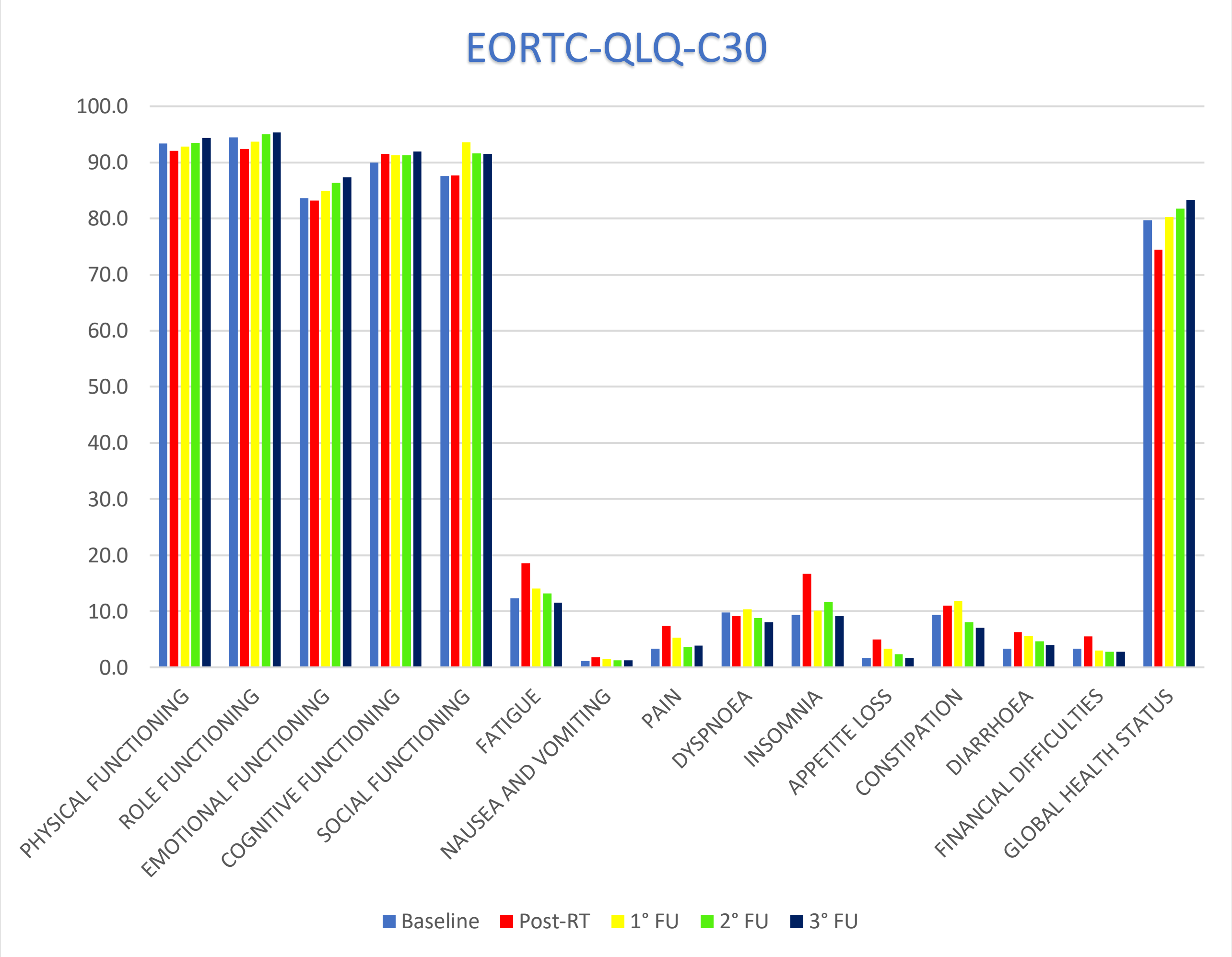

EORTC-QLQ-C30	Mean (%) ± SD	p value (vs Baseline)
PHYSICAL FUNCTIONING		
- BASELINE	93.3 ± 12.99	-
- POST-RT	92.1 ± 13.59	0.014
- 1° FU	92.9 ± 12.75	0.675
- 2° FU	93.4 ± 12.06	0.810
- 3° FU	94.3 ± 11.22	0.220
ROLE FUNCTIONING		
- BASELINE	94.4 ± 14.70	-
- POST-RT	92.4 ± 16.71	0.033
- 1° FU	93.8 ± 13.93	0.033
- 2° FU	95.0 ± 12.06	0.340
- 3° FU	95.3 ± 12.33	0.054
EMOTIONAL FUNCTIONING		
- BASELINE	83.6 ± 18.33	-
- POST-RT	83.2 ± 18.76	0.617
- 1° FU	85.0 ± 18.54	0.142
- 2° FU	86.3 ± 18.29	0.010
- 3° FU	87.3 ± ±18.25	0.0002

COGNITIVE FUNCTIONING		
- BASELINE	90.0 ± 14.98	-
- POST-RT	91.5 ± 13.29	0.197
- 1° FU	91.3 ± 14.11	0.230
- 2° FU	86.3 ± 18.29	0.342
- 3° FU	92.0 ± 12.86	0.107
SOCIAL FUNCTIONING		
- BASELINE	86.7 ± 25.18	-
- POST-RT	87.7 ± 23.53	0.889
- 1° FU	93.6 ± 13.9	0.714
- 2° FU	91.7 ± 20.31	0.016
- 3° FU	91.5 ± 20.99	0.016
FATIGUE		
- BASELINE	12.3 ± 17.83	-
- POST-RT	18.5 ± 22.28	0.0012
- 1° FU	14.0 ± 20.26	0.226
- 2° FU	13.2 ± 20.79	0.810
- 3° FU	11.5 ± 18.69	0.401
NAUSEA AND VOMITING		
- BASELINE	1.10 ± 5.47	-
- POST-RT	1.80 ± 5.76	0.226
- 1° FU	1.40 ± 6.35	0.417
- 2° FU	1.30 ± 6.16	0.658
- 3° FU	1.30 ± 6.16	0.658
PAIN		
- BASELINE	3.30 ± 13.81	-
- POST-RT	7.3 ± 14.85	0.009
- 1° FU	5.3 ± 12.73	0.033
- 2° FU	3.7 ± 11.98	0.565
- 3° FU	3.8 ± 14.18	0.320
DYSPNOEA		
- BASELINE	9.8 ± 20.52	-
- POST-RT	9.2 ± 20.01	0.638
- 1° FU	10.3 ± 21.16	0.674
- 2° FU	8.8 ± 19.60	0.320
- 3° FU	8.0 ± 18.27	0.320
INSOMNIA		
- BASELINE	9.3 ± 19.58	-
- POST-RT	16.7 ± 24.39	0.0008
- 1° FU	10.2 ± 19.23	0.529
- 2° FU	11.6 ± 20.71	0.158
- 3° FU	9.1 ± 18.39	0.842
APPETITE LOSS		
- BASELINE	1.7 ± 7.29	-
- POST-RT	5.0 ± 13.71	0.005
- 1° FU	3.3 ± 11.11	0.025
- 2° FU	2.3 ± 8.54	0.158
- 3° FU	1.7 ± 7.29	1
CONSTIPATION		
- BASELINE	9.3 ± 21.75	-
- POST-RT	11.0 ± 19.56	0.447
- 1° FU	11.8 ± 37.67	0.509
- 2° FU	8.0 ± 20.17	0.249
- 3° FU	7.0 ± 18.53	0.188
DIARRHOEA		
- BASELINE	3.3 ± 10.04	-
- POST-RT	6.3 ± 14.75	0.064

- 1° FU<br>- 2° FU<br>- 3° FU	5.7 ± 13.23<br>4.7 ± 12.55<br>4.0 ± 14.43	0.197<br>0.348<br>0.671
FINANCIAL DIFFICULTIES<br>- BASELINE<br>- POST-RT<br>- 1° FU<br>- 2° FU<br>- 3° FU	 3.3 ± 12.97<br>5.5 ± 16.25<br>3.0 ± 10.69<br>2.8 ± 9.78<br>2.8 ± 9.78	 -<br>0.042<br>0.657<br>0.469<br>0.469
GLOBAL HEALTH STATUS<br>- BASELINE<br>- POST-RT<br>- 1° FU<br>- 2° FU<br>- 3° FU	 79.7 ± 17.25<br>74.4 ± 18.79<br>80.3 ± 17.21<br>81.8 ± 16.19<br>83.3 ± 16.31	 -<br>0.00008<br>0.631<br>0.072<br>0.001

Table S2 – ICIQ-SF scores

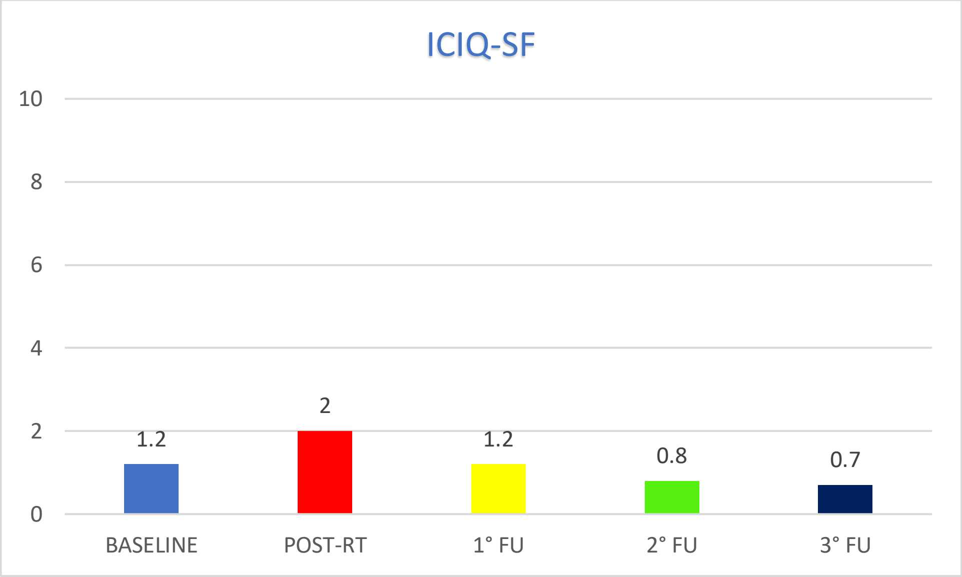

ICIQ-SF	Mean ± SD	p value (vs Baseline)
- BASELINE	1.2 ± 2.8	-
- POST-RT	2.0 ± 3.4	0.0008
- 1° FU	1.2 ± 2.6	0.66
- 2° FU	0.8 ± 2.3	0.72
- 3° FU	0.7 ± 2.1	0.36
-		

Table S3 – IIEF-5 scores

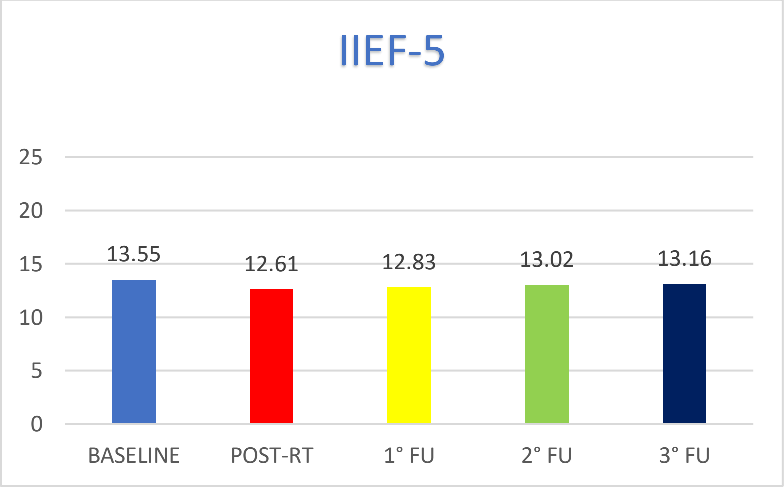

IIEF-5	Mean ± SD	p value (vs Baseline)
- BASELINE	13.55 ± 9.24	-
- POST-RT	12.61 ± 9.28	0.0001
- 1° FU	12.83 ± 9.14	0.075
- 2° FU	12.92 ± 9.25	0.477
- 3° FU	13.02 ± 9.31	0.682

Table S4 – EORTC-QLQ-PR25 scores

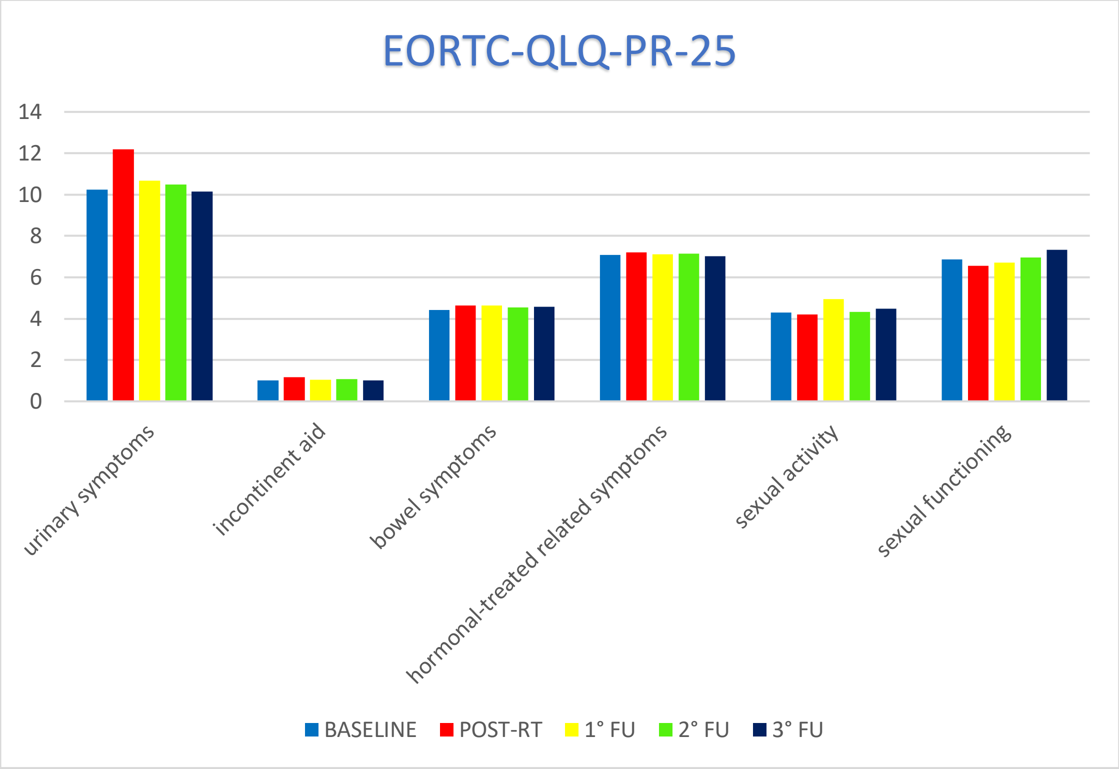

EORTC-QLQ-PR-25	Mean ± SD	p value (vs Baseline)
URINARY SYMPTOMS		
- BASELINE	10.23 ± 2.24	-
- POST-RT	12. 19 ± 3.52	0.0001
- 1° FU	10.66 ± 2.31	0.0067
- 2° FU	10.48 ± 2.84	0.46
- 3° FU	10.16 ± 2.36	0.38
INCONTINENT AID		
- BASELINE	1.02 ± 0.14	-
- POST-RT	1.16 ± 0.60	0.013
- 1° FU	1.03 ± 0.17	0.32
- 2° FU	1.07 ± 0.36	0.32
- 3° FU	1.02 ± 0.14	1
BOWEL SYMPTOMS		
- BASELINE	4.42 ± 0.74	-
- POST-RT	4.63 ± 0.87	0.007
- 1° FU	4.63 ± 6.16	0.03
- 2° FU	4.53 ± 1.03	0.19
- 3° FU	4.56 ± 0.96	0.06
HORMONAL-TREATED RELATED SYMPTOMS		
- BASELINE	7.09 ± 1.31	-
- POST-RT	7.19 ± 1.53	0.46

- 1° FU	7.12 ± 1.53	0.82
- 2° FU	7.13 ± 1.50	0.77
- 3° FU	7.01 ± 1.23	0.44
SEXUAL ACTIVITY		
- BASELINE	4.3 ± 1.62	-
- POST-RT	4.2 ± 1.50	0.32
- 1° FU	4.93 ± 6.16	0.30
- 2° FU	4.34 ± 1.60	0.72
- 3° FU	4.48 ± 1.59	0.16
SEXUAL FUNCTIONING		
- BASELINE	6.85 ± 2.65	-
- POST-RT	6.54 ± 2.63	0.16
- 1° FU	6.71 ± 2.61	0.49
- 2° FU	6.97 ± 2.81	0.62
- 3° FU	7.33 ± 3.13	0.31

Table S5 – EPIC-26 scores

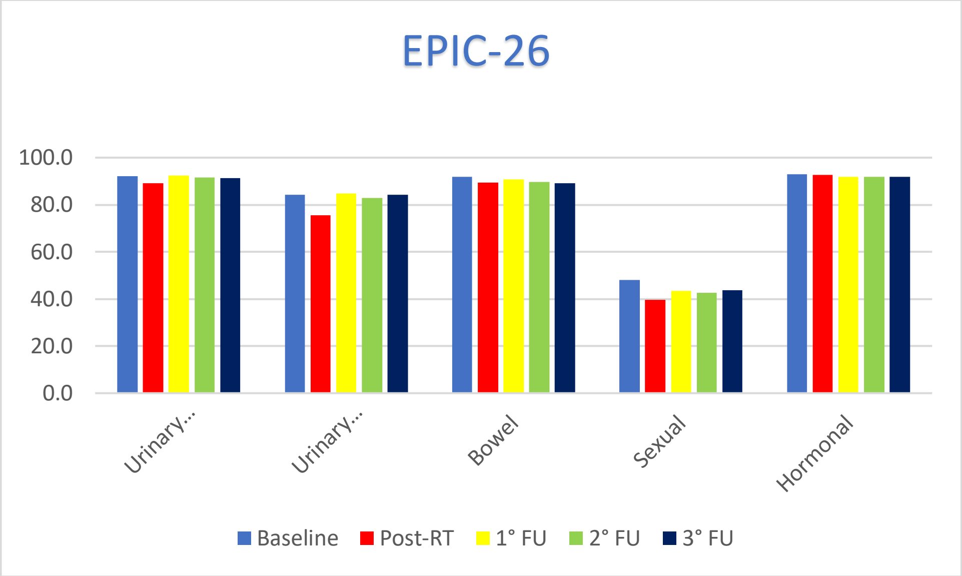

EPIC-26	Mean ± SD	p value (vs Baseline)
**URINARY INCONTINENCE**		
- BASELINE	92.1 + 13.2	-
- POST-RT	89.0 + 16.1	0.011
- 1° FU	92.3 + 12.6	0.646
- 2° FU	91.5 + 13.8	0.826
- 3° FU	91.4 + 14.1	0.689
**URINARY IRRITATIVE/OBSTRUCTIVE**		
- BASELINE	84.1 + 17.2	-
- POST-RT	75.6 + 21.2	0.0002
- 1° FU	84.8 + 14.1	0.881
- 2° FU	82.9 + 18.1	0.424
- 3° FU	84.2 + 15.4	0.984
**BOWEL**		
- BASELINE	91.9 + 15.8	-
- POST-RT	89.3 + 17.3	0.089
- 1° FU	90.8 + 14.9	0.222
- 2° FU	89.8 + 17.0	0.134
- 3° FU	89.0 + 18.6	0.054
**SEXUAL**		
- BASELINE	48.1 + 29.9	-
- POST-RT	39.6 + 28.4	0.0001
- 1° FU	43.4 + 29.8	0.0003
- 2° FU	42.7 + 29.4	0.005
- 3° FU	43.7 + 28.9	0.02
**HORMONAL**		
- BASELINE	93.0 + 10.9	-
- POST-RT	92.6 + 11.7	0.779
- 1° FU	91.9 + 10.8	0.150
- 2° FU	91.9 + 10.6	0.226
- 3° FU	91.8 + 10.3	0.164

Table S6 – IPSS scores

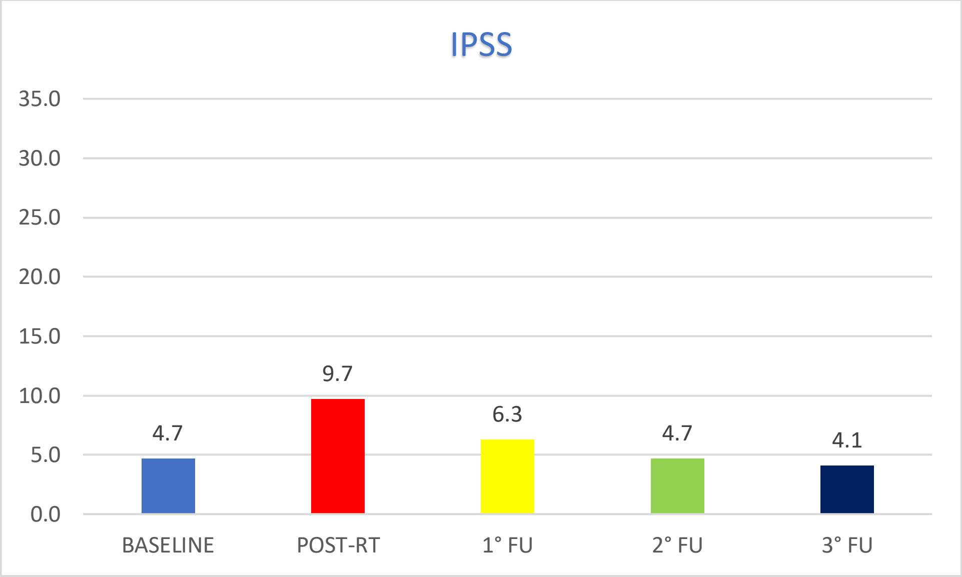

IPSS	Mean ± SD	p value (vs Baseline)
- BASELINE	4.7 ± 3.2	-
- POST-RT	9.7 ± 6.7	0.0001
- 1° FU	6.3 ± 4.6	0.0001
- 2° FU	4.7 ± 3.3	0.810
- 3° FU	4.7 ± 3.0	0.818
